# The complete chloroplast genome sequence of *Afzelia xylocarpa*

**DOI:** 10.1080/23802359.2020.1715857

**Published:** 2020-01-21

**Authors:** Jinfeng Zhang, Yunqing Li, Sokh Heng, Kimsrim Seab, Yi Wang

**Affiliations:** a Laboratory of Forest Plant Cultivation and Utilization, Yunnan Academy of Forestry, Kunming, Yunnan, People’s Republic of China;; b Institute of Forest and Wildlife Research and Development Forestry Administration, Phnom, Cambodia

**Keywords:** *Afzelia xylocarpa*, chloroplast, Illumina sequencing, phylogenetic analysis

## Abstract

The first complete chloroplast genome (cpDNA) sequence of *Afzelia xylocarpa* was determined from Illumina HiSeq pair-end sequencing data in this study. The cpDNA is 159,115 bp in length, contains a large single-copy region (LSC) of 88,164 bp and a small single-copy region (SSC) of 19,495 bp, which were separated by a pair of inverted repeats (IR) regions of 25,748 bp. The genome contains 128 genes, including 83 protein-coding genes, 8 ribosomal RNA genes, and 37 transfer RNA genes. The overall GC content of the whole genome is 36.1%, and the corresponding values of the LSC, SSC, and IR regions are 33.7%, 29.6%, and 42.6%, respectively. Further phylogenomic analysis showed that *A. xylocarpa, Tamarindus indica*, and *Crudia harmsiana* are clustered in a clade in the Detarioideae subfamily.

*Afzelia xylocarpa* is the species within the subfamily Detarioideae in Fabaceae. It is mainly distributed in Thailand, Myanmar, Malay Peninsula, and cultivated in Yunnan, Guangdong, Guangxi, Hainan, Taiwan of China (Pakkad et al. 2009). The extract of *Afzelia* genus plants showed antioxidant (Akinpelu et al. 2010), antidiabetic (Oyedemi et al. 2011), antimicrobial, and anti-inflammatory activities (Akah et al. 2007). In China, *A. xylocarpa* is widely used as a folk medicine to treat inflammation and eye diseases, sore throat, and food poisoning (Cai et al. 2018). Therefore, *A. xylocarpa* has potential medicinal value. However, there has been no genomic study on *A. xylocarpa.*

Herein, we reported and characterized the complete *A. xylocarpa* plastid genome. The GenBank accession number is MN823693. One *A. xylocarpa* individual (specimen number: 201907023) was collected from Puwen, Yunnan Province of China (23°31′49ʺN, 101°37′41ʺE). The specimen is stored at Yunnan Academy of Forestry Herbarium, Kunming, China, and the accession number is ZJFEP112. DNA was extracted from its fresh leaves using DNA Plantzol Reagent (Invitrogen, Carlsbad, CA, USA).

Paired-end reads were sequenced using Illumina HiSeq system (Illumina, San Diego, CA). In total, about 22.7 million high-quality clean reads were generated with adaptors trimmed. Aligning, assembly, and annotation were conducted by CLC *de novo* assembler (CLC Bio, Aarhus, Denmark), BLAST, GeSeq (Tillich et al. 2017), and GENEIOUS v 11.0.5 (Biomatters Ltd, Auckland, New Zealand). To confirm the phylogenetic position of *A. xylocarpa*, other four species of *Detarioideae* subfamily from NCBI were aligned using MAFFT v.7 (Katoh and Standley 2013). The auto algorithm in the MAFFT alignment software was used to align the eight complete genome sequences and G-INS-i algorithm was used to align the partial complex sequences. The maximum likelihood (ML) bootstrap analysis was conducted using RAxML (Stamatakis 2006); bootstrap probability values were calculated from 1000 replicates. *Stryphnodendron adstringens* (MN196294) and *Parkia javanica* (KX852442) were served as the out-group.

The complete *A. xylocarpa* plastid genome is a circular DNA molecule with the length of 159,115 bp, containing a large single-copy region (LSC) of 88,164 bp and a small single-copy region (SSC) of 19,495 bp, which were separated by a pair of inverted repeats (IR) regions of 25,748 bp. The overall GC content of the whole genome is 36.1%, and the corresponding values of the LSC, SSC, and IR regions are 33.7%, 29.6%, and 42.6%, respectively. The plastid genome contained 128 genes, including 83 protein-coding genes, 8 ribosomal RNA genes, and 37 transfer RNA genes. Phylogenetic analysis showed that *A. xylocarpa, Tamarindus indica*, and *Crudia harmsiana* clustered in a unique clade in the Detarioideae subfamily (Figure 1). The determination of complete plastid genome sequences provided new molecular data to illuminate the Detarioideae subfamily evolution.

**Figure 1.**
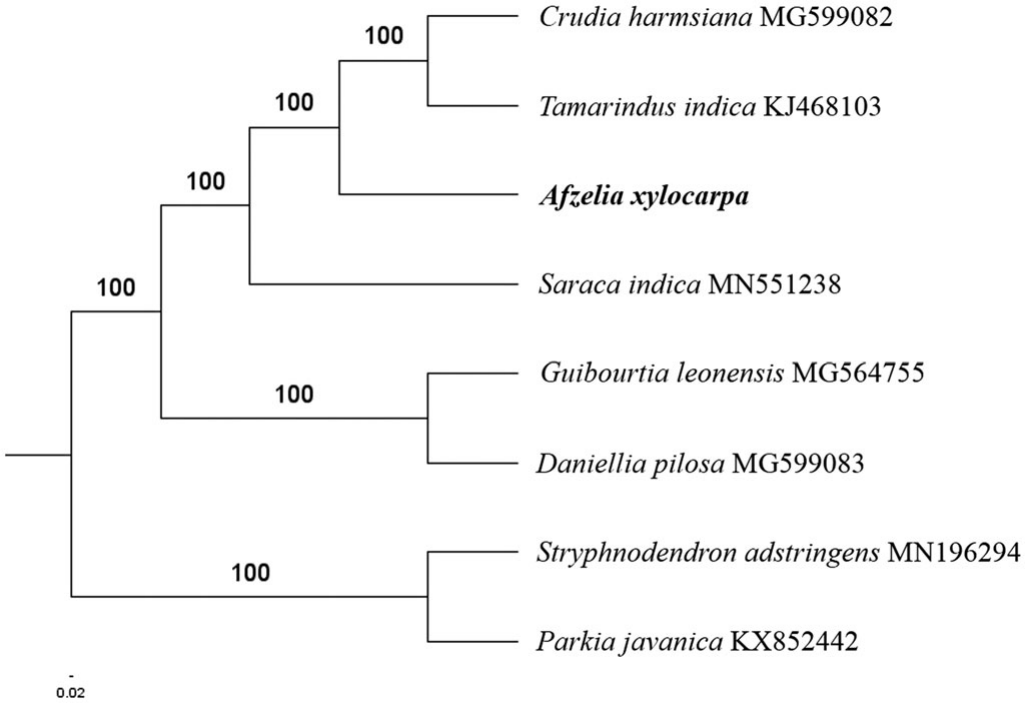
The maximum-likelihood tree based on the six chloroplast genomes of *Detarioideae* subfamily. The bootstrap value based on 1000 replicates is shown on each node.
